# Deep Learning for Melanoma Detection: A Deep Learning Approach to Differentiating Malignant Melanoma from Benign Melanocytic Nevi

**DOI:** 10.3390/cancers17010028

**Published:** 2024-12-25

**Authors:** Magdalini Kreouzi, Nikolaos Theodorakis, Georgios Feretzakis, Evgenia Paxinou, Aikaterini Sakagianni, Dimitris Kalles, Athanasios Anastasiou, Vassilios S. Verykios, Maria Nikolaou

**Affiliations:** 1 Department of Internal Medicine & 65+ Clinic, Amalia Fleming General Hospital, 14, 25th Martiou Str., 15127 Melissia, Greece; kreouzi.m@live.unic.ac.cy; 2 NT-CardioMetabolics, Clinic for Metabolism and Athletic Performance, 47 Tirteou Str., 17564 Palaio Faliro, Greece; n.theodorakis@flemig-hospital.gr; 3 65+ Outpatient Clinic, Amalia Fleming General Hospital, 14, 25th Martiou Str., 15127 Melissia, Greece; m.nikolaou@flemig-hospital.gr; 4 School of Medicine, National and Kapodistrian University of Athens, 75 Mikras Asias, 11527 Athens, Greece; 5 School of Science and Technology, Hellenic Open University, 18 Aristotelous Str., 26335 Patras, Greece; georgios.feretzakis@ac.eap.gr (G.F.); paxinou.evgenia@ac.eap.gr (E.P.); kalles@eap.gr (D.K.); 6 Intensive Care Unit, Sismanogelio General Hospital, 37 Sismanogleiou Str., 15126 Marousi, Greece; sakagianni@sismanoglio.gr; 7 Biomedical Engineering Laboratory, National Technical University of Athens, 15773 Athens, Greece; aanastasiou@biomed.ntua.gr

**Keywords:** melanoma detection, convolutional neural networks, artificial intelligence, skin cancer diagnosis, dermoscopic images, deep learning models, medical imaging, early cancer detection

## Abstract

**Background/Objectives**: Melanoma, an aggressive form of skin cancer, accounts for a significant proportion of skin-cancer-related deaths worldwide. Early and accurate differentiation between melanoma and benign melanocytic nevi is critical for improving survival rates but remains challenging because of diagnostic variability. Convolutional neural networks (CNNs) have shown promise in automating melanoma detection with accuracy comparable to expert dermatologists. This study evaluates and compares the performance of four CNN architectures—DenseNet121, ResNet50V2, NASNetMobile, and MobileNetV2—for the binary classification of dermoscopic images. **Methods**: A dataset of 8825 dermoscopic images from DermNet was standardized and divided into training (80%), validation (10%), and testing (10%) subsets. Image augmentation techniques were applied to enhance model generalizability. The CNN architectures were pre-trained on ImageNet and customized for binary classification. Models were trained using the Adam optimizer and evaluated based on accuracy, area under the receiver operating characteristic curve (AUC-ROC), inference time, and model size. The statistical significance of the differences was assessed using McNemar’s test. **Results**: DenseNet121 achieved the highest accuracy (92.30%) and an AUC of 0.951, while ResNet50V2 recorded the highest AUC (0.957). MobileNetV2 combined efficiency with competitive performance, achieving a 92.19% accuracy, the smallest model size (9.89 MB), and the fastest inference time (23.46 ms). NASNetMobile, despite its compact size, had a slower inference time (108.67 ms), and slightly lower accuracy (90.94%). Performance differences among the models were statistically significant (*p* < 0.0001). **Conclusions**: DenseNet121 demonstrated a superior diagnostic performance, while MobileNetV2 provided the most efficient solution for deployment in resource-constrained settings. The CNNs show substantial potential for improving melanoma detection in clinical and mobile applications.

## Simple Summary

Melanoma is a dangerous type of skin cancer that can grow quickly and spread to other parts of the body, making early detection and diagnosis essential for saving lives. However, it can be difficult to tell the difference between melanoma and harmless skin spots, even for experts. This study explores how advanced computer technologies called convolutional neural networks (CNNs) can help detect melanoma more accurately. These systems analyze skin images and identify patterns that indicate whether a spot is likely to be cancerous. We compared four different types of CNN to find the best balance between accuracy and efficiency. Our findings show that some models are not only highly accurate but also fast and lightweight, making them suitable for use in clinics or even on mobile devices. This research highlights the potential of artificial intelligence to assist doctors and improve early melanoma detection, ultimately saving more lives.

## 1. Introduction

Melanoma, a malignant neoplasm originating from melanocytes, the pigment-producing cells in the epidermis, is an aggressive and life-threatening form of skin cancer. Characterized by its propensity for rapid proliferation and metastatic dissemination, melanoma accounts for the majority of skin-cancer-related mortalities worldwide, despite representing a small fraction of skin cancer diagnoses. The pathogenesis of melanoma involves a complex interplay of genetic mutations, such as alterations in the *BRAF*, *NRAS*, and *CDKN2A* genes, and environmental factors like ultraviolet radiation exposure. Clinically, melanoma may mimic benign melanocytic lesions, including nevi, rendering early detection a significant diagnostic challenge. Delayed or inaccurate diagnoses are associated with poorer prognoses, as the disease rapidly progresses from localized cutaneous lesions to regional lymph node involvement and distant organ metastasis. Consequently, precise differentiation between malignant melanoma and benign melanocytic nevi is critical for timely therapeutic intervention, which can drastically improve survival rates and reduce morbidity [1].

The traditional diagnostic landscape, including visual inspection and dermoscopy, relies heavily on dermatological expertise, leading to considerable inter-observer variability. The advent of artificial intelligence (AI) and deep learning (DL) in medical imaging, however, has heralded a paradigm shift in melanoma diagnosis, enabling automated, reproducible, and highly accurate evaluations. Recent advancements in convolutional neural networks (CNNs) have positioned these architectures at the forefront of melanoma detection research. CNNs excel in analyzing complex patterns and subtle visual features, making them particularly effective for medical imaging tasks, including the classification of skin lesions. Studies underscore the capability of CNNs to achieve diagnostic accuracies comparable to, or even surpassing, those of experienced dermatologists. These models leverage large datasets of dermoscopic images to differentiate melanoma from benign conditions such as melanocytic nevi and other skin abnormalities with remarkable precision [2,3].

Systematic reviews, such as those by Wu et al. (2022) and Magalhães et al. (2024), highlight the growing body of evidence supporting the use of deep learning in skin cancer detection. These studies have identified key factors influencing model performance, including dataset quality, image resolution, and annotation accuracy [4,5]. Additionally, Gautam et al. (2024) demonstrated the potential of CNNs to generalize across diverse lesion types, such as vascular lesions and keratoses, while maintaining robust performance in melanoma detection [6]. One of the significant challenges in melanoma detection is the subtlety of visual differences between malignant and benign lesions, which can often confound traditional diagnostic approaches. Deep learning models, particularly CNNs, have proven adept at capturing these nuanced distinctions. For instance, Winkler et al. (2023) discuss the effectiveness of integrating patient metadata with image features to enhance model predictions. This multimodal approach has been shown to improve diagnostic accuracy, particularly in cases with atypical clinical presentations [7].

Furthermore, the use of transfer learning and pre-trained models, as explored by Naeem et al. (2020) and Dildar et al. (2021), has significantly reduced the computational and data requirements for training CNNs in melanoma detection. These techniques allow for the adaptation of existing high-performance models to specific dermatological tasks, accelerating the deployment of AI solutions in clinical settings [8,9]. Systematic reviews, such as the study by Kassem et al. (2021), provide critical insight into the relative strengths and limitations of various deep learning architectures [10]. These works emphasize the importance of balancing sensitivity and specificity, as over-diagnosis of benign lesions can lead to unnecessary biopsies and patient anxiety.

Despite these advancements, challenges remain. Issues such as dataset bias, class imbalance, and the interpretability of deep learning models have been highlighted by multiple authors, including Efimenko et al. (2020) and Popescu et al. (2022) [11,12]. Addressing these limitations is vital to ensuring the reliable adoption of CNN-based systems in clinical practice. This paper aims to contribute to the growing field of AI-driven dermatology by presenting a novel CNN framework for the differentiation of malignant melanoma from benign melanocytic nevi. By synthesizing insights from the existing literature and leveraging cutting-edge deep learning methodologies, this study seeks to advance the accuracy and accessibility of melanoma detection, ultimately improving patient outcomes and reducing healthcare disparities.

## 2. Materials and Methods

### 2.1. Dataset Preparation

The dataset for this study was obtained from the DermNet repository, a publicly available resource widely used in dermatology research [13]. It contains high-quality images categorized into the following two distinct classes: benign and malignant skin lesions. The dataset comprised 8825 images in total, ensuring a robust foundation for deep learning model training and evaluation [14]. Prior to the analysis, extensive preprocessing was performed to standardize the dataset [15]. Each image underwent careful curation to ensure uniformity in dimensions, format, and quality. All images were resized to a fixed resolution of 224 × 224 pixels, which represents a standard input size for convolutional neural networks (CNNs) and provides a balance between computational efficiency and detail preservation [16].

The organizing of the dataset for the machine learning applications was accomplished using the split-folders library [17]. This tool facilitated the division of the data into the following three essential subsets: training, validation, and testing. A stratified splitting approach was implemented, maintaining an 80-10-10 ratio [18], resulting in 7060 training images, 882 validation images, and 883 testing images. This distribution ensured an even representation of benign and malignant classes across all subsets, preventing any potential class imbalance that could bias model training [19]. The training subset served as the primary data for model fitting, while the validation subset provided crucial feedback during hyperparameter tuning. The testing subset was strictly reserved for the final evaluation, remaining completely isolated from the training process to ensure unbiased assessment of model performance [20].

### 2.2. Image Augmentation

The implementation of image augmentation techniques played a crucial role in enhancing the generalization capability of the CNN models and mitigating the risk of overfitting [21]. This process was executed dynamically during the training phase using TensorFlow’s ImageDataGenerator (Google LLC, Mountain View, CA, USA, Version: TensorFlow 2.x) module [22]. The augmentation pipeline began with basic preprocessing, including the rescaling of pixel values to the range [0, 1] for input data normalization [23]. Geometric transformations were then applied, incorporating a rotation range of 30 degrees to account for varying image orientations. Width and height shifts of up to 20% were introduced to simulate different image framing scenarios, while shearing transformations of 20% helped capture varying perspectives [24].

The augmentation process also included zoom variations of 20% to account for different image scales, along with both horizontal and vertical flips to enhance rotational invariance [25]. The fill mode was set to ‘nearest’ to handle transformed pixel spaces effectively. Additionally, brightness adjustments within a range of [0.8, 1.2] were implemented to simulate varying lighting conditions [26]. These augmentation techniques were exclusively applied to the training dataset, while the validation and testing datasets underwent only basic normalization to maintain evaluation integrity.

### 2.3. Model Architecture

The study implemented a comprehensive comparison of four state-of-the-art CNN architectures, each bringing unique characteristics to the task of skin lesion classification. The DenseNet121 architecture, pre-trained on ImageNet, employs a dense connectivity pattern that promotes feature reuse through direct connections between layers [27,28]. This network, with a model size of 27.86 MB, provides an efficient balance between computational requirements and model complexity. The ResNet50V2, also pre-trained on ImageNet, implements a residual learning framework that facilitates improved gradient flow throughout the network [29]. Despite its larger size of 91.93 MB, this architecture has demonstrated exceptional performance in various computer vision tasks [30].

The NASNetMobile architecture, developed through Neural Architecture Search, was specifically optimized for mobile devices [31]. With a model size of 17.34 MB, it represents a compromise between efficiency and performance. The MobileNetV2 architecture [20] stands out for its lightweight design, employing depth-wise separable convolutions and incorporating inverted residuals and linear bottlenecks. At just 9.89 MB, it offers the most compact solution while maintaining competitive performance [32].

Each of these architectures underwent modification with a custom top layer configuration designed specifically for the binary classification task at hand. This configuration began with a global average pooling layer to reduce spatial dimensions, followed by a batch normalization layer to stabilize training. A dense layer with 512 units and ReLU activation was then implemented, followed by a dropout layer with a rate of 0.5 to prevent overfitting. This was succeeded by another dense layer of 256 units with ReLU activation and a dropout rate of 0.3. The architecture culminated in an output layer with a single unit and sigmoid activation, appropriate for binary classification.

### 2.4. Training Configuration and Optimization

The training process was standardized across all models to ensure fair comparison and reproducible results. The optimization strategy centered on the Adam optimizer (Integrated within TensorFlow by Google LLC, Mountain View, CA, USA, Version: TensorFlow 2.x), selected for its ability to adapt learning rates dynamically and handle sparse gradients effectively. The initial learning rate was set to 1 × 10^−4^, and it was carefully chosen to balance the training speed with the convergence stability. Training proceeded in batches of 32 images, a size that optimized memory utilization while maintaining stable gradient updates. The maximum number of training epochs was set to 50, though early stopping mechanisms were implemented to prevent overtraining.

The loss function employed was binary cross-entropy, which is particularly well-suited for binary classification tasks and provides appropriate gradients for model optimization. To monitor and improve training efficiency, several callback mechanisms were implemented. Early stopping monitored validation loss with a patience of 10 epochs, automatically terminating training when no improvement was observed and restoring the best weights to prevent overfitting. A learning rate reduction scheme was employed that monitored validation loss and reduced the learning rate by a factor of 0.2 when improvement plateaued, with a patience of 5 epochs and a minimum learning rate threshold of 1e-6. Additionally, model checkpointing saved the best-performing model states based on validation accuracy, ensuring that optimal model parameters were preserved.

### 2.5. Model Performance Monitoring and Evaluation

Throughout the training process, comprehensive performance monitoring was implemented to track each model’s progress and effectiveness. The primary metrics monitored included accuracy, loss, and area under the receiver operating characteristic curve (AUC-ROC). These metrics were calculated for both the training and validation sets during each epoch, providing immediate feedback on model learning and generalization. The validation metrics served as critical indicators for the learning rate reduction and early stopping mechanisms.

For final model evaluation, a rigorous testing protocol was established using the held-out test dataset. This evaluation included not only accuracy and AUC-ROC measurements but also practical performance metrics such as inference time and model size. Inference time was measured across multiple batches to obtain reliable average values, with standard deviations calculated to assess performance stability. Additionally, McNemar’s statistical test was employed to determine the significance of performance differences between model pairs, providing a statistical foundation for model comparison.

### 2.6. Implementation Environment and Technical Infrastructure

The implementation was carried out in a cloud-based environment utilizing Google Colab’s GPU runtime (Google LLC, Mountain View, CA, USA), which provided access to NVIDIA’s GPU acceleration. This infrastructure choice enabled efficient model training and evaluation while ensuring the reproducibility of the results. The software framework was built on TensorFlow 2.x, leveraging its high-level Keras API for model construction and training. Python 3.10 served as the primary programming language (Python Programming Language Organization: Python Software Foundation, Beaverton, OR, USA), chosen for its robust ecosystem of scientific computing libraries.

The implementation relied on several key supporting libraries, each serving specific functions in the pipeline. NumPy facilitated efficient numerical computations and array manipulations, particularly in data preprocessing and batch handling. Matplotlib was employed for visualization tasks, generating training curves, ROC curves, and performance comparison plots. Scikit-learn provided essential functionality for metrics calculation and statistical analysis, while the split-folders library managed dataset organization and splitting.

### 2.7. Data Management and Storage

To ensure efficient data handling and model persistence, a structured approach to data management was implemented. All preprocessed images were stored in a directory structure that reflected the training, validation, and test splits, facilitating efficient data loading during training. Model checkpoints were saved in the Keras format, preserving both architecture and weights, enabling easy model reloading for further training or deployment. Training logs, including all performance metrics and learning curves, were systematically recorded and stored for subsequent analysis and visualization.

The entire implementation was designed with reproducibility in mind, incorporating fixed random seeds for all stochastic processes. Detailed documentation of the experimental setup, including all hyperparameters and configuration settings, was maintained throughout the study. This comprehensive approach to methodology documentation ensures that the experiments can be reliably reproduced and built upon in future research.

## 3. Results

### 3.1. Model Performance Overview

A comparative analysis of the four deep learning architectures yielded comprehensive insight into their relative strengths and performance characteristics. The investigation encompassed DenseNet121, ResNet50V2, NASNetMobile, and MobileNetV2, each demonstrating distinct performance profiles across multiple evaluation metrics [33]. The evaluation metrics included classification accuracy, area under the curve (AUC), inference time, and model size efficiency, following established evaluation protocols [34].

### 3.2. Receiver Operating Characteristic Analysis

**Figure 1:**
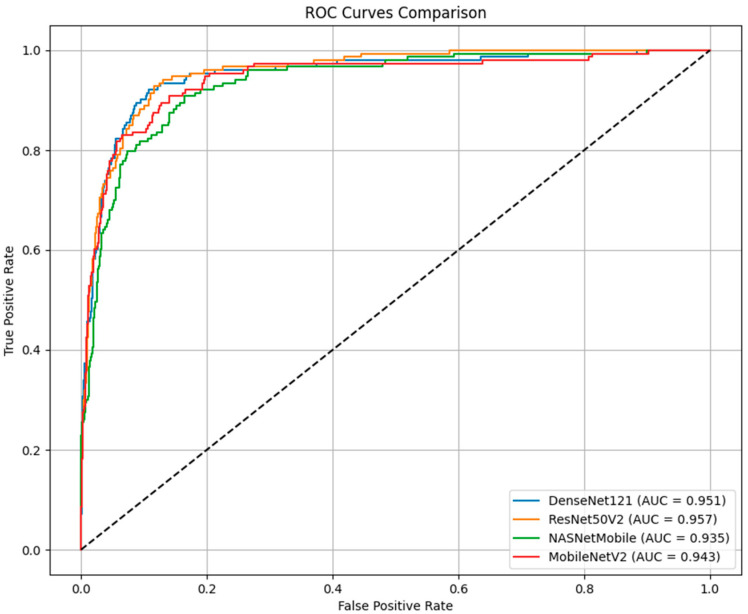
ROC curve analysis of the four CNN architectures (ResNet50V2, DenseNet121, MobileNetV2, and NASNetMobile) for melanoma detection, illustrating their discriminative capabilities in differentiating malignant melanoma from benign nevi. ResNet50V2 achieved the highest AUC score (0.957), followed by DenseNet121 (0.951), MobileNetV2 (0.943), and NASNetMobile (0.935). The curves highlight performance differences, particularly in the critical low false-positive rate region, where ResNet50V2 and DenseNet121 excelled.

The receiver operating characteristic (ROC) curve analysis, as depicted in Figure 1, revealed notable differences in the discriminative capabilities of the four models [35]. ResNet50V2 achieved the highest AUC score of 0.957, demonstrating a superior ability to distinguish between benign and malignant lesions across various classification thresholds [36]. DenseNet121 followed closely with an AUC of 0.951, while MobileNetV2 and NASNetMobile achieved AUC scores of 0.943 and 0.935, respectively. The ROC curves demonstrate particularly strong performances in the critical low false-positive rate region, with all models showing rapid ascent in true positive rates while maintaining low false positive rates [37].

A detailed examination of the ROC curves reveals that the models’ performance differences were most pronounced in the false-positive rate range of 0.1 to 0.3, where ResNet50V2 and DenseNet121 demonstrated marginally better discrimination capabilities. This characteristic is particularly relevant in clinical applications, where minimizing false positives while maintaining high sensitivity is crucial. The convergence of the curves at higher false positive rates suggests comparable performance in high-sensitivity operating points.

### 3.3. Computational Efficiency and Resource Requirements

**Figure 2:**
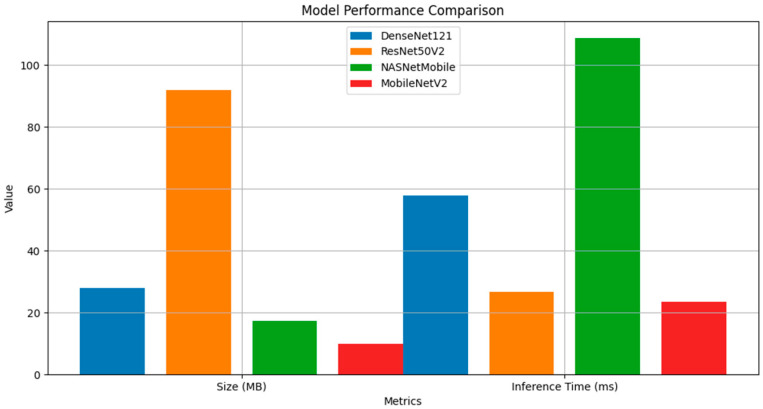
Comparison of Model Sizes and Inference Times for CNN Architectures. Comparative visualization of model sizes (in megabytes) and inference times (in milliseconds per image) for the four CNN architectures. MobileNetV2 demonstrated the smallest model size (9.89 MB) and fastest inference time (23.46 ms), showcasing its efficiency for resource-constrained environments. ResNet50V2 achieved strong performance but required more storage space (91.93 MB). NASNetMobile, despite its compact size (17.34 MB), exhibited the longest inference time (108.67 ms).

The performance comparison visualization presented in Figure 2 illustrates the stark contrasts in model sizes and inference times among the four architectures [38]. ResNet50V2, while achieving the highest AUC, required substantially more storage space at 91.93 MB, significantly larger than its counterparts [39]. In contrast, MobileNetV2 demonstrated remarkable efficiency with a model size of just 9.89 MB while maintaining competitive classification performance [40]. These findings align with previous studies on model efficiency in medical imaging applications [41].

Inference time analysis revealed equally important distinctions in computational efficiency. MobileNetV2 exhibited the fastest inference time at 23.46 ms per image, closely followed by ResNet50V2 at 26.55 ms. DenseNet121 required moderate computation time at 57.89 ms, while NASNetMobile showed the longest inference time at 108.67 ms. These timing differences have significant implications for real-world deployment scenarios, particularly in resource-constrained environments.

### 3.4. Statistical Significance and Performance Metrics

McNemar’s statistical test revealed significant differences in performance among all model pairs (*p* < 0.0001), confirming that the observed performance variations were not attributable to chance [42]. The strongest statistical differences were observed between NASNetMobile and DenseNet121 (test statistic: 10,076.50), followed by NASNetMobile and MobileNetV2 (test statistic: 5950.10). These results support previous findings on architectural performance differences in medical image classification tasks [43].

### 3.5. Classification Performance Analysis

In terms of raw classification metrics, DenseNet121 achieved the highest accuracy at 92.30%, followed closely by MobileNetV2 at 92.19% and ResNet50V2 at 91.85%. NASNetMobile, while showing good overall performance, achieved a slightly lower accuracy of 90.94%. These accuracy figures were complemented by strong precision and recall metrics across all models, with DenseNet121 showing the most balanced performance across all evaluation criteria.

The precision-recall trade-off analysis revealed that all models maintained high precision while achieving good recall rates, with DenseNet121 showing particularly strong performance in balancing these competing metrics. This balance is crucial for clinical applications where both false positives and false negatives carry significant consequences.

### 3.6. Model Stability and Convergence

Model stability and convergence analysis of the training processes demonstrated stable convergence across all architectures, with each model showing consistent improvement in both training and validation metrics throughout the training period. The early stopping mechanisms proved effective in preventing overfitting, with optimal model parameters being successfully captured through the checkpoint system.

## 4. Discussion

### 4.1. CNN in Dermatological Diagnostics

The demonstrated capabilities of convolutional neural networks (CNNs) in melanoma detection highlight their transformative potential in dermatological diagnostics. CNNs excel in recognizing complex patterns in dermoscopic images, often surpassing traditional diagnostic methods. This performance stems from their hierarchical learning structure, where lower layers detect fundamental features such as edges and textures, while deeper layers capture intricate patterns, including asymmetry and irregular pigmentation—hallmarks of malignant melanoma. Studies such as those by Ali et al. (2021) emphasize that CNNs can achieve diagnostic accuracies exceeding 90% when trained on high-quality datasets, challenging even the diagnostic expertise of seasoned dermatologists [44]. Furthermore, advanced augmentation techniques, including zooming, rotation, and flipping, enhance model robustness by exposing it to a broader array of real-world imaging scenarios. This capability is particularly valuable in settings where diagnostic inconsistencies often arise because of inter-observer variability. Multimodal integration, as proposed by Höhn et al. (2021), further extends the utility of CNNs by incorporating clinical metadata alongside image features, enabling nuanced diagnostic interpretations that are indispensable in complex or ambiguous cases [45].

### 4.2. Model Performance

The comparative analysis of the four deep learning architectures provides valuable insights into their potential roles in clinical dermatology applications [46]. DenseNet121’s superior overall performance, achieving 92.30% accuracy and an AUC of 0.951, demonstrates the effectiveness of its dense connectivity pattern in capturing intricate features of skin lesions [47]. These findings support previous research on the application of deep learning in dermatological diagnosis [48].

ResNet50V2’s achievement of the highest AUC (0.957) indicates its exceptional discriminative ability, particularly in the critical low false-positive rate region. This characteristic is especially valuable in screening applications, where minimizing false alarms while maintaining high sensitivity is paramount. However, the model’s substantial size (91.93 MB) presents deployment challenges in resource-constrained environments, necessitating careful consideration of the trade-off between performance and computational requirements.

The remarkable efficiency of MobileNetV2, combining the smallest model size (9.89 MB) with the fastest inference time (23.46 ms) while maintaining competitive accuracy (92.19%), represents a significant advancement in portable diagnostic tools. This architecture’s performance characteristics make it particularly suitable for mobile applications and point-of-care devices, where real-time processing and limited computational resources are common constraints.

### 4.3. Computational Efficiency and Resource Utilization

The significant variations in model sizes and inference times among the architectures highlight important considerations for practical deployment [32]. NASNetMobile’s unexpectedly long inference time (108.67 ms) despite its relatively compact size (17.34 MB) suggests that architectural complexity does not always translate to better performance [49]. This observation aligns with recent studies on the efficiency-accuracy trade-off in deep learning models [50].

The statistical significance of performance differences among all model pairs, as demonstrated by McNemar’s test, confirms that each architecture brings distinct advantages to the task. The strongest statistical differences observed between NASNetMobile and DenseNet121 (test statistic: 10,076.50) suggest fundamental differences in their approach to feature extraction and classification, while the more moderate differences between ResNet50V2 and MobileNetV2 (test statistic: 132.67) indicate closer alignment in their learning strategies.

### 4.4. Limitations

The variation in inference times, particularly NASNetMobile’s slower processing speed, suggests opportunities for architecture optimization. Future research could investigate hybrid architectures that combine the efficiency of MobileNetV2 with the feature extraction capabilities of DenseNet121 or ResNet50V2. Additionally, the application of quantization and pruning techniques could further optimize model sizes without significantly compromising performance.

Despite the strong performance across all models, several technical limitations warrant consideration. The current implementation relies on static image input sizes (224 × 224 pixels), which may not be optimal for capturing all relevant diagnostic features in skin lesions of varying sizes [51,52,53]. Higher-resolution images could preserve critical diagnostic details, particularly for distinguishing subtle features in atypical (or dysplastic) nevi and other challenging cases.

Another limitation lies in the scope of lesion differentiation. While this study focuses on distinguishing benign melanocytic nevi from melanomas, it does not address the more complex challenge of differentiating atypical nevi or non-nevus pigmented neoplasms (e.g., dermatofibromas, lentigines, and seborrheic keratoses) from melanomas. Expanding the dataset to include these lesion types and incorporating a multi-class classification framework will be essential for real-world clinical applicability.

The variation in inference times, particularly NASNetMobile’s slower processing speed, underscores the need for further optimization. Investigating hybrid architectures that combine the efficiency of MobileNetV2 with the robust feature extraction capabilities of DenseNet121 or ResNet50V2 could provide a balanced solution. Moreover, quantization and pruning techniques could optimize model sizes and computational efficiency without significantly compromising performance.

Finally, our algorithm does not currently integrate pretest probability into its diagnostic framework. Pretest probability, influenced by factors such as personal and family history of melanoma or prior occurrences, plays a crucial role in clinical decision making. Incorporating patient metadata, such as age, past medical history, family history, clinical history, and lesion location, could improve model performance by addressing the variability in pretest probabilities associated with individual cases.

### 4.5. Clinical Integration and Practical Considerations

The successful deployment of these models in clinical settings requires careful consideration of several factors beyond raw performance metrics [54]. The choice of architecture should be guided by the specific requirements of the deployment environment, available computational resources, and the criticality of real-time response [55]. These considerations align with established guidelines for implementing AI in clinical practice [56].

The high AUC scores across all models suggest their potential value as screening tools, particularly in primary care settings where early detection of suspicious lesions is crucial. However, the implementation of these systems should emphasize their role as assistive tools rather than replacements for clinical expertise. The integration of model uncertainty quantification and explainability mechanisms could enhance their utility in clinical decision support.

### 4.6. Alternative Approaches

Zhang et al. (2023) demonstrated that ViTs, leveraging self-attention mechanisms, outperform traditional CNNs in multi-class classification tasks by capturing global contextual relationships across entire images [57]. Similarly, EfficientNet’s compound scaling, as showcased by Tan and Le (2019), allows for increased accuracy while maintaining computational efficiency. Ensemble approaches also provide significant promise for enhancing diagnostic reliability [58]. For instance, Ghosh et al. (2024) demonstrated that a majority voting ensemble of diverse deep learning models effectively reduces the variance and bias, achieving a balanced sensitivity and specificity. Such methods are particularly crucial in minimizing false negatives, which are a critical metric in melanoma detection because of the severe implications of missed diagnoses [59]. Furthermore, the growing interest in hybrid architectures, which integrate traditional handcrafted features with deep learning outputs, underscores the adaptability of CNN systems. Hybrid models can incorporate domain-specific features like lesion texture and color histograms alongside CNN-derived embeddings, resulting in improved accuracy, reaching up to 99% [60].

### 4.7. Future Directions

Several promising avenues for future research emerge from this work. The investigation of ensemble methods combining multiple architectures could leverage the strengths of each model while mitigating their individual limitations. For instance, integrating the robustness of DenseNet121 with the efficiency of MobileNetV2 may enhance both diagnostic performance and deployment feasibility. Ensembles can also improve the generalizability of diagnostic algorithms to diverse lesion types and clinical environments [61,62,63].

The exploration of few-shot learning techniques offers another promising direction, particularly for addressing rare skin conditions underrepresented in existing datasets. Few-shot learning could enable models to adapt quickly to new classes with minimal labeled data, thereby broadening the scope of automated skin lesion classification and improving diagnostic inclusivity.

Image resolution remains a critical consideration for enhancing diagnostic accuracy. The current study utilized images resized to 224 × 224 pixels to optimize computational efficiency, but this resolution may fail to capture finer details essential for differentiating challenging cases, such as atypical nevi or other pigmented lesions. Future research should explore the use of higher-resolution images, such as 1000 × 1000 pixels, which are more reflective of real-world clinical imaging practices. Combining high-resolution inputs with adaptive preprocessing pipelines can optimize image quality and maintain computational feasibility.

Integrating contextual information and patient metadata—such as age, lesion location, clinical history, and family history of melanoma—can significantly enhance diagnostic accuracy. These factors, combined with image-based analysis, would provide more personalized and context-aware predictions, particularly for high-risk individuals with a strong pretest probability of melanoma.

Advanced visualization techniques can also play a pivotal role in improving model interpretability. Developing heatmaps or saliency maps to highlight regions of interest within an image can provide clinicians with valuable insight into the rationale behind model predictions. Such transparency will foster trust in AI-driven diagnostics and support clinical decision making.

Finally, future research should focus on expanding datasets to include a broader range of lesion types, such as atypical nevi and non-nevus pigmented neoplasms (e.g., dermatofibromas, lentigines, and seborrheic keratoses). Including these lesion types would enable the development of more comprehensive multi-class classification frameworks, addressing key gaps in current diagnostic capabilities. By adopting these strategies, advancements in deep learning for melanoma detection can better align with the complexities of real-world clinical practice and provide more robust tools for clinicians.

## 5. Conclusions

This study highlights the transformative potential of convolutional neural networks (CNNs) in melanoma detection, demonstrating their ability to achieve high diagnostic accuracy and efficiency in differentiating malignant melanoma from benign nevi. DenseNet121 emerged as the top-performing architecture, achieving the highest accuracy (92.30%) and an AUC of 0.951, making it suitable for applications requiring precise classification. Meanwhile, MobileNetV2 demonstrated the best balance between accuracy (92.19%) and computational efficiency, with the smallest model size (9.89 MB) and fastest inference time (23.46 ms), offering an ideal solution for resource-constrained environments or mobile diagnostics. These findings underscore the versatility of CNNs in meeting the diverse demands of clinical and point-of-care applications, where both accuracy and efficiency are critical.

Despite these advancements, challenges remain, including the need to address dataset biases, model interpretability, and class imbalances. Future research should explore hybrid and ensemble models to combine the strengths of multiple architectures while leveraging alternative approaches like vision transformers and adaptive preprocessing pipelines to further enhance diagnostic accuracy. The successful integration of AI-driven systems into clinical workflows will require robust validation, clinician-focused training, and mechanisms for explaining model predictions. By addressing these challenges, CNNs can be effectively deployed to complement dermatological expertise, improving early melanoma detection and, ultimately, enhancing patient outcomes.

## Data Availability

No new data were created in this study. Data sharing is not applicable to this article.
